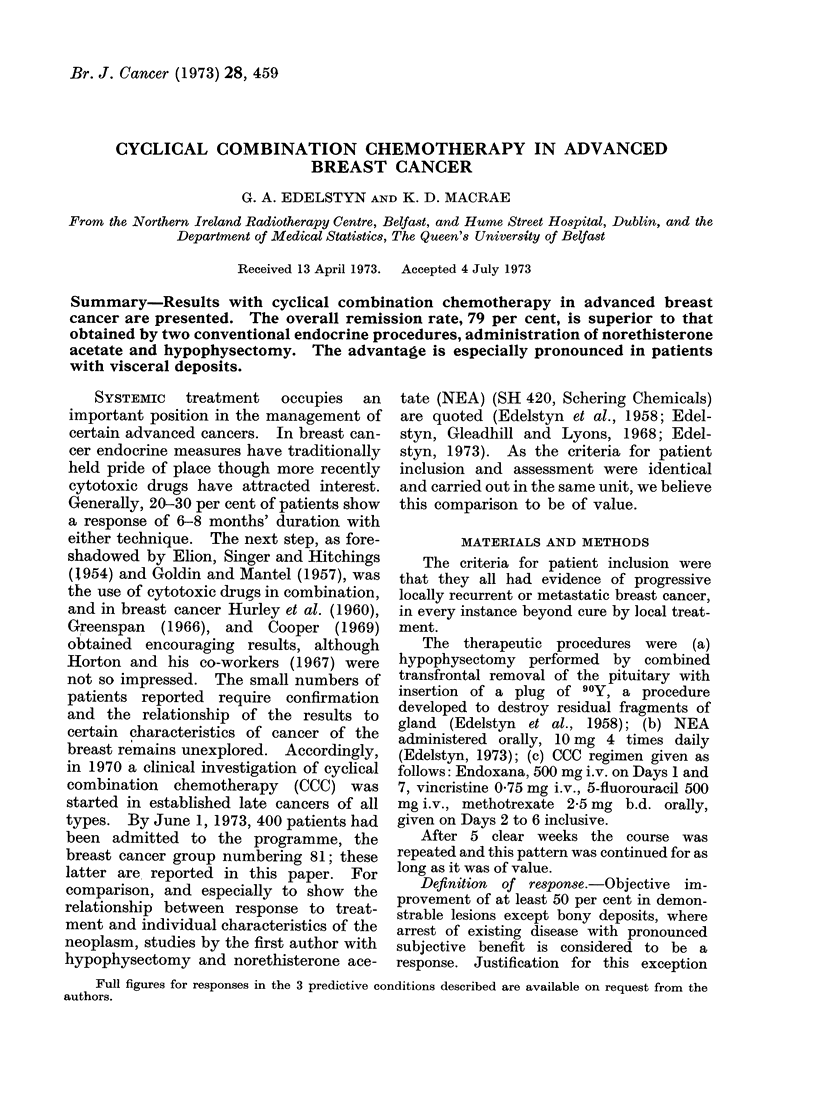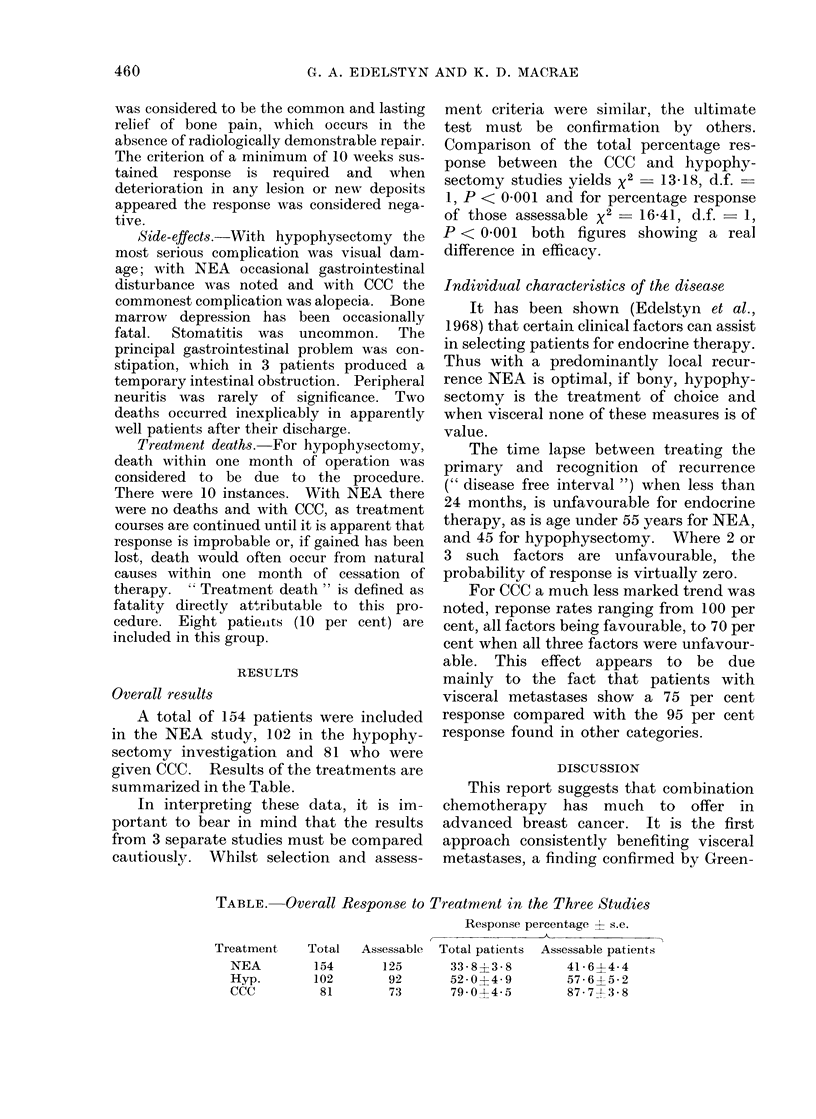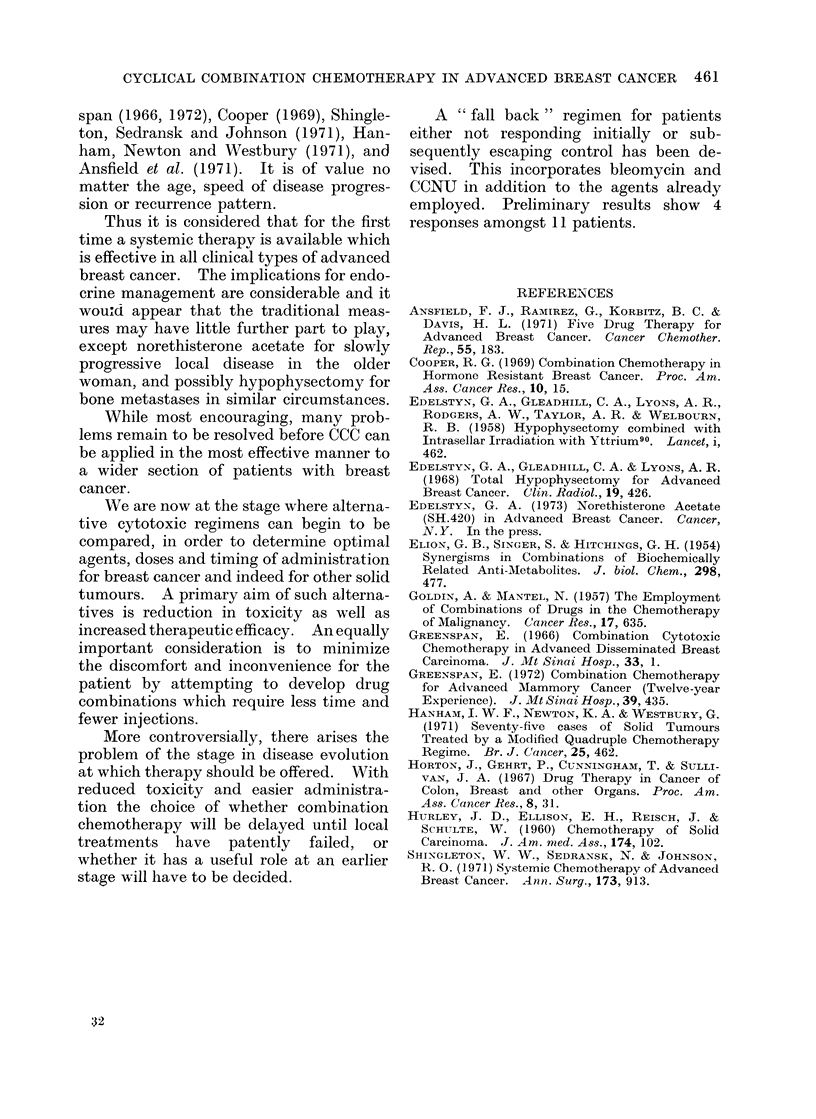# Cyclical Combination Chemotherapy in Advanced Breast Cancer

**DOI:** 10.1038/bjc.1973.172

**Published:** 1973-11

**Authors:** G. A. Edelstyn, K. D. Macrae

## Abstract

Results with cyclical combination chemotherapy in advanced breast cancer are presented. The overall remission rate, 79 per cent, is superior to that obtained by two conventional endocrine procedures, administration of norethisterone acetate and hypophysectomy. The advantage is especially pronounced in patients with visceral deposits.


*Br. J. Cancer* (1973) **28**, 459

### CYCLICAL COMBINATION CHEMOTHERAPY IN ADVANCED BREAST CANCER

G. A. EDELSTYN AND K. D. MACRAE

*From the Northern Ireland Radiotherapy Centre, Belfast, and Hume Street Hospital, Dublin, and the Department of Medical Statistics, The Queen's University of Belfast*

Received 13 April 1973. Accepted 4 July 1973

**Summary—Results with cyclical combination chemotherapy in advanced breast cancer are presented. The overall remission rate, 79 per cent, is superior to that obtained by two conventional endocrine procedures, administration of norethisterone acetate and hypophysectomy. The advantage is especially pronounced in patients with visceral deposits.**

SYSTEMIC treatment occupies an important position in the management of certain advanced cancers. In breast cancer endocrine measures have traditionally held pride of place though more recently cytotoxic drugs have attracted interest. Generally, 20–30 per cent of patients show a response of 6–8 months' duration with either technique. The next step, as foreshadowed by Elion, Singer and Hitchings (1954) and Goldin and Mantel (1957), was the use of cytotoxic drugs in combination, and in breast cancer Hurley *et al.* (1960), Greenspan (1966), and Cooper (1969) obtained encouraging results, although Horton and his co-workers (1967) were not so impressed. The small numbers of patients reported require confirmation and the relationship of the results to certain characteristics of cancer of the breast remains unexplored. Accordingly, in 1970 a clinical investigation of cyclical combination chemotherapy (CCC) was started in established late cancers of all types. By June 1, 1973, 400 patients had been admitted to the programme, the breast cancer group numbering 81; these latter are reported in this paper. For comparison, and especially to show the relationship between response to treatment and individual characteristics of the neoplasm, studies by the first author with hypophysectomy and norethisterone acetate (NEA) (SH 420, Schering Chemicals) are quoted (Edelstyn *et al.*, 1958; Edelstyn, Gleadhill and Lyons, 1968; Edelstyn, 1973). As the criteria for patient inclusion and assessment were identical and carried out in the same unit, we believe this comparison to be of value.

### MATERIALS AND METHODS

The criteria for patient inclusion were that they all had evidence of progressive locally recurrent or metastatic breast cancer, in every instance beyond cure by local treatment.

The therapeutic procedures were (a) hypophysectomy performed by combined transfrontal removal of the pituitary with insertion of a plug of $^{90}Y$, a procedure developed to destroy residual fragments of gland (Edelstyn *et al.*, 1958); (b) NEA administered orally, 10 mg 4 times daily (Edelstyn, 1973); (c) CCC regimen given as follows: Endoxana, 500 mg i.v. on Days 1 and 7, vincristine 0·75 mg i.v., 5-fluorouracil 500 mg i.v., methotrexate 2·5 mg b.d. orally, given on Days 2 to 6 inclusive.

After 5 clear weeks the course was repeated and this pattern was continued for as long as it was of value.

*Definition of response.*—Objective improvement of at least 50 per cent in demonstrable lesions except bony deposits, where arrest of existing disease with pronounced subjective benefit is considered to be a response. Justification for this exception

Full figures for responses in the 3 predictive conditions described are available on request from the authors.

was considered to be the common and lasting relief of bone pain, which occurs in the absence of radiologically demonstrable repair. The criterion of a minimum of 10 weeks sustained response is required and when deterioration in any lesion or new deposits appeared the response was considered negative.

*Side-effects.*—With hypophysectomy the most serious complication was visual damage; with NEA occasional gastrointestinal disturbance was noted and with CCC the commonest complication was alopecia. Bone marrow depression has been occasionally fatal. Stomatitis was uncommon. The principal gastrointestinal problem was constipation, which in 3 patients produced a temporary intestinal obstruction. Peripheral neuritis was rarely of significance. Two deaths occurred inexplicably in apparently well patients after their discharge.

*Treatment deaths.*—For hypophysectomy, death within one month of operation was considered to be due to the procedure. There were 10 instances. With NEA there were no deaths and with CCC, as treatment courses are continued until it is apparent that response is improbable or, if gained has been lost, death would often occur from natural causes within one month of cessation of therapy. " Treatment death " is defined as fatality directly attributable to this procedure. Eight patients (10 per cent) are included in this group.

### RESULTS

### *Overall results*

A total of 154 patients were included in the NEA study, 102 in the hypophysectomy investigation and 81 who were given CCC. Results of the treatments are summarized in the Table.

In interpreting these data, it is important to bear in mind that the results from 3 separate studies must be compared cautiously. Whilst selection and assessment criteria were similar, the ultimate test must be confirmation by others. Comparison of the total percentage response between the CCC and hypophysectomy studies yields $\chi^2 = 13{\cdot}18$, d.f. $= 1$, $P < 0{\cdot}001$ and for percentage response of those assessable $\chi^2 = 16{\cdot}41$, d.f. $= 1$, $P < 0{\cdot}001$ both figures showing a real difference in efficacy.

### *Individual characteristics of the disease*

It has been shown (Edelstyn *et al.*, 1968) that certain clinical factors can assist in selecting patients for endocrine therapy. Thus with a predominantly local recurrence NEA is optimal, if bony, hypophysectomy is the treatment of choice and when visceral none of these measures is of value.

The time lapse between treating the primary and recognition of recurrence (" disease free interval ") when less than 24 months, is unfavourable for endocrine therapy, as is age under 55 years for NEA, and 45 for hypophysectomy. Where 2 or 3 such factors are unfavourable, the probability of response is virtually zero.

For CCC a much less marked trend was noted, reponse rates ranging from 100 per cent, all factors being favourable, to 70 per cent when all three factors were unfavourable. This effect appears to be due mainly to the fact that patients with visceral metastases show a 75 per cent response compared with the 95 per cent response found in other categories.

### DISCUSSION

This report suggests that combination chemotherapy has much to offer in advanced breast cancer. It is the first approach consistently benefiting visceral metastases, a finding confirmed by Green-

TABLE.—*Overall Response to Treatment in the Three Studies*

| Treatment | Total | Assessable | Response percentage ± s.e. Total patients | Assessable patients |
|---|---|---|---|---|
| NEA | 154 | 125 | 33·8±3·8 | 41·6±4·4 |
| Hyp. | 102 | 92 | 52·0±4·9 | 57·6±5·2 |
| CCC | 81 | 73 | 79·0±4·5 | 87·7±3·8 |

span (1966, 1972), Cooper (1969), Shingleton, Sedransk and Johnson (1971), Hanham, Newton and Westbury (1971), and Ansfield *et al.* (1971). It is of value no matter the age, speed of disease progression or recurrence pattern.

Thus it is considered that for the first time a systemic therapy is available which is effective in all clinical types of advanced breast cancer. The implications for endocrine management are considerable and it would appear that the traditional measures may have little further part to play, except norethisterone acetate for slowly progressive local disease in the older woman, and possibly hypophysectomy for bone metastases in similar circumstances.

While most encouraging, many problems remain to be resolved before CCC can be applied in the most effective manner to a wider section of patients with breast cancer.

We are now at the stage where alternative cytotoxic regimens can begin to be compared, in order to determine optimal agents, doses and timing of administration for breast cancer and indeed for other solid tumours. A primary aim of such alternatives is reduction in toxicity as well as increased therapeutic efficacy. An equally important consideration is to minimize the discomfort and inconvenience for the patient by attempting to develop drug combinations which require less time and fewer injections.

More controversially, there arises the problem of the stage in disease evolution at which therapy should be offered. With reduced toxicity and easier administration the choice of whether combination chemotherapy will be delayed until local treatments have patently failed, or whether it has a useful role at an earlier stage will have to be decided.

A " fall back " regimen for patients either not responding initially or subsequently escaping control has been devised. This incorporates bleomycin and CCNU in addition to the agents already employed. Preliminary results show 4 responses amongst 11 patients.

### REFERENCES

Ansfield, F. J., Ramirez, G., Korbitz, B. C. & Davis, H. L. (1971) Five Drug Therapy for Advanced Breast Cancer. *Cancer Chemother. Rep.*, **55**, 183.

Cooper, R. G. (1969) Combination Chemotherapy in Hormone Resistant Breast Cancer. *Proc. Am. Ass. Cancer Res.*, **10**, 15.

Edelstyn, G. A., Gleadhill, C. A., Lyons, A. R., Rodgers, A. W., Taylor, A. R. & Welbourn, R. B. (1958) Hypophysectomy combined with Intrasellar Irradiation with Yttrium$^{90}$. *Lancet*, i, 462.

Edelstyn, G. A., Gleadhill, C. A. & Lyons, A. R. (1968) Total Hypophysectomy for Advanced Breast Cancer. *Clin. Radiol.*, **19**, 426.

Edelstyn, G. A. (1973) Norethisterone Acetate (SH.420) in Advanced Breast Cancer. *Cancer, N.Y.* In the press.

Elion, G. B., Singer, S. & Hitchings, G. H. (1954) Synergisms in Combinations of Biochemically Related Anti-Metabolites. *J. biol. Chem.*, **298**, 477.

Goldin, A. & Mantel, N. (1957) The Employment of Combinations of Drugs in the Chemotherapy of Malignancy. *Cancer Res.*, **17**, 635.

Greenspan, E. (1966) Combination Cytotoxic Chemotherapy in Advanced Disseminated Breast Carcinoma. *J. Mt Sinai Hosp.*, **33**, 1.

Greenspan, E. (1972) Combination Chemotherapy for Advanced Mammory Cancer (Twelve-year Experience). *J. Mt Sinai Hosp.*, **39**, 435.

Hanham, I. W. F., Newton, K. A. & Westbury, G. (1971) Seventy-five cases of Solid Tumours Treated by a Modified Quadruple Chemotherapy Regime. *Br. J. Cancer*, **25**, 462.

Horton, J., Gehrt, P., Cunningham, T. & Sullivan, J. A. (1967) Drug Therapy in Cancer of Colon, Breast and other Organs. *Proc. Am. Ass. Cancer Res.*, **8**, 31.

Hurley, J. D., Ellison, E. H., Reisch, J. & Schulte, W. (1960) Chemotherapy of Solid Carcinoma. *J. Am. med. Ass.*, **174**, 102.

Shingleton, W. W., Sedransk, N. & Johnson, R. O. (1971) Systemic Chemotherapy of Advanced Breast Cancer. *Ann. Surg.*, **173**, 913.